# Genetic risk score correlates with immune profile and risk of HCC and cirrhosis development in Hispanics with MASLD

**DOI:** 10.1016/j.jhepr.2025.101508

**Published:** 2025-07-02

**Authors:** Siyu Fu, Zwier M.A. Groothuismink, Domingo Balderramo, Angelo Z. Mattos, Lisia Hoppe, Enrique Carrera, Javier Diaz-Ferrer, Jhon Prieto, Jesus M. Banales, Marco Arrese, Bettina E. Hansen, Andre Boonstra, José D. Debes

**Affiliations:** 1 Department of Gastroenterology and Hepatology, Erasmus MC University Medical Center, Rotterdam, The Netherlands; 2 Hospital Privado Universitario de Córdoba, Instituto Universitario de Ciencias Biomédicas de Córdoba, Córdoba, Argentina; 3 Graduate Program in Medicine: Hepatology, Federal University of Health Sciences of Porto Alegre, Porto Alegre, Brazil; 4 Department of Gastroenterology and Hepatology, University of Passo Fundo, Passo Fundo, Rio Grande do Sul, Brazil; 5 Hospital Especialidades Eugenio Espejo, Universidad San Francisco de Quito, Quito, Ecuador; 6 Hospital Edgardo Rebagliati Martins, Facultad de Medicina Humana, Universidad de San Martin de Pores, Lima, Peru; 7 Centro de Enfermedades Hepáticas y Digestivas (CEHYD), Bogota, Colombia; 8 Department of Liver and Gastrointestinal Diseases, Biogipuzkoa Health Research Institute, Donostia University Hospital, University of the Basque Country (UPV/EHU), CIBERehd, Ikerbasque, San Sebastian, Spain; 9 Department of Biochemistry and Genetics, School of Sciences, University of Navarra, Pamplona, Spain; 10 Departamento de Gastroenterología, Facultad de Medicina, Pontificia Universidad Católica de Chile, Santiago, Chile; 11 Toronto Centre for Liver Disease, University Health Network, University of Toronto, Toronto, Ontario, Canada; 12 Department of Epidemiology, Biostatistics, Erasmus University Medical Center, Rotterdam, The Netherlands; 13 Department of Medicine, University of Minnesota, Minneapolis, MN, USA

**Keywords:** *PNPLA3*, *TM6SF2*, *MBOAT7*, *HSD17B13*, Genetic risk score, Cytokine, MASLD, HCC, Cirrhosis

## Abstract

**Background & Aims:**

Genetic risk score and immune dysregulations have been separately associated with the development of hepatocellular carcinoma (HCC) and cirrhosis in the context of metabolic dysfunction-associated steatotic liver disease (MASLD). Latin America has the highest prevalence of MASLD worldwide. However, the relationship between genetic risk scores, immune dysregulation, and MASLD has not been explored.

**Methods:**

We assessed SNPs of *PNPLA3* rs738409, *TM6SF2* rs58542926, *MBOAT7* rs641738, and *HSD17B13* rs72613567 in samples from a cohort of 972 Latin American individuals (HCC = 267, non-HCC = 705). The four SNPs were later combined into a genetic risk score and calculated in patients with MASLD (cirrhotic HCC = 133, cirrhosis = 242, non-cirrhotic liver disease (NCLD) = 113). A total of 28 cytokines were analyzed in a subgroup of these samples (cirrhotic HCC = 107, cirrhosis = 111).

**Results:**

At an individual level, only *PNPLA3* GG genotype was associated with a significantly increased risk of MASLD-related HCC (odds ratio [OR]: 2.805, 95% CI: 1.083–7.264, *p* = 0.034) and cirrhosis (OR: 6.873, 95% CI: 3.293–14.35, *p* <0.001). When the four SNPs were combined into a genetic risk score, patients with a score of 6–8 had higher odds of MASLD-related HCC (OR: 3.603, 95% CI: 1.008–12.88, *p* = 0.049) and cirrhosis (OR: 13.12, 95% CI: 2.270–75.76, *p* = 0.004) compared with those with a score of 0–2. Cytokine profiles differed by genetic risk score in MASLD-related HCC and cirrhosis. Patients with HCC with high scores had lower levels of interferon-gamma and CCL8 (false discovery rate <0.05), whereas patients with cirrhosis with high scores showed higher matrix metallopeptidase 2 (MMP2) levels (false discovery rate <0.05).

**Conclusions:**

In Latin America, genetic risk score 6–8 in patients is strongly associated with an increased risk of MASLD-related HCC and cirrhosis. Additionally, patients with HCC and cirrhosis showed distinct immune profiles across high and low genetic risk score groups.

**Impact and implications:**

The prevalence of metabolic dysfunction-associated steatotic liver disease (MASLD)-related hepatocellular carcinoma (HCC) and cirrhosis is rising, with Hispanics having the highest MASLD rates. However, large-scale studies examining the association between genetic risk score, immune profiles, and the progression of MASLD-related HCC and cirrhosis are still lacking. In our study, we found that patients with MASLD-related HCC and cirrhosis who had higher genetic risk score were more likely to show higher odds ratios compared with those with lower genetic risk score. Additionally, genetic risk scores were found to be associated with immune profiles, as reflected by cytokine levels. These findings could assist clinicians in identifying high-risk groups of patients with MASLD-related HCC and cirrhosis and provide valuable insights into the potential immune changes in these individuals.

## Highlights

• Genetic risk score strongly correlates with MASLD-related HCC and cirrhosis.
• Genetic risk score influences the immune profile in MASLD-related HCC and cirrhosis.
• MASLD-related HCC and cirrhosis show distinct immune activity based on genetic risk score.

## Graphical abstract

**Figure:**
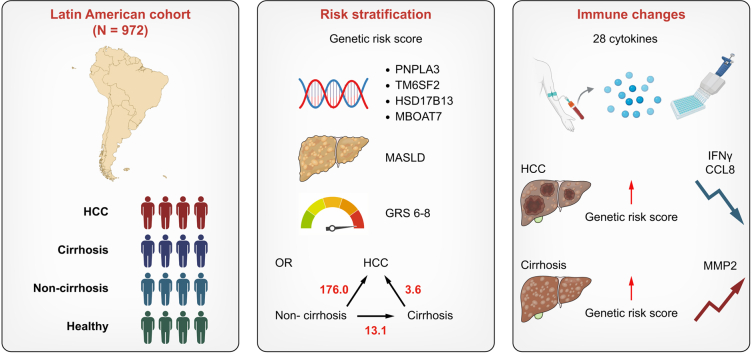

## Introduction

Metabolic dysfunction-associated steatotic liver disease (MASLD), previously referred to as non-alcoholic fatty liver disease (NAFLD), has rapidly become the most prevalent liver disease worldwide, affecting approximately 38% of the global population, mainly driven by increasing rates of obesity and type 2 diabetes mellitus.[1], [2], [3] MASLD encompasses a spectrum of liver conditions, including metabolic dysfunction-associated steatohepatitis (MASH), MASLD-related cirrhosis and MASLD-related hepatocellular carcinoma (HCC).^4^ Although MASLD-related HCC occurs at a lower incidence compared with HCC from other etiologies, the high prevalence of MASLD contributes to a significant number of HCC cases.^5^^,^^6^ These HCCs sometimes occur before the development of cirrhosis and are frequently diagnosed at advanced stages outside routine surveillance programs, leading to poor outcomes.^6^ The absence of reliable HCC risk-stratification tools in MASLD highlights the urgent need for novel biomarkers and risk-assessment strategies to identify high-risk groups enabling detection of HCC at early stages. Hispanics have some of the highest MASLD rates globally and have recently experienced a marked increase in the proportion of HCC cases attributable to MASLD, representing an at-risk but understudied population.[7], [8], [9], [10]

Genetic factors are pivotal in MASLD development and progression, with genetic susceptibility assessments offering the potential to refine HCC risk stratification.^11^ Genome-wide association studies have identified several single-nucleotide polymorphisms (SNPs) strongly associated with MASLD.^11^ Indeed, SNPs in *PNPLA3*,^12^ *MBOAT7*,^13^ and *TM6SF2*,^14^ are linked to increased liver fat accumulation and to an increased risk of MASLD, fibrosis, and HCC.^11^ Conversely, *HSD17B13* variants demonstrate a protective effect against cirrhosis and HCC.^15^^,^^16^ Combining multiple SNPs into a genetic risk score (GRS) provides a more accurate assessment of genetic susceptibility compared with individual SNPs and can help identify patients at high risk of developing HCC.[17], [18], [19], [20] These SNPs and GRS have primarily been tested in European populations, with alcohol-related liver disease (ALD) or HCV as the main etiologies, and not in Latin American populations in which MASLD prevalence is particularly high.^7^^,^^8^ Therefore, assessing the performance of SNPs in lipid metabolism-related genes and their combined effects through GRS in stratifying MASLD-related liver disease severity and progression within Latin American populations is crucial.

The SNPs currently known to be associated with MASLD are primarily within genes involved in lipid metabolism. The specific mechanisms related to these lipid-related modifications and HCC are, however, unclear. HCC is an inflammation-driven cancer, as most cases occur in the setting of chronic immune reaction related to a viral or metabolic process. Studies have suggested the involvement of various pro-inflammatory cytokines in lipid metabolism in metabolic diseases, including cancer.^21^ Although some studies have evaluated the relation of *PNPLA3* with IL-6 and IL-8 modulation in MASLD *in vitro*, no study has evaluated the association between HCC genetic predisposition measured by GRS and immune marker dysregulation in relation to these mutations and risk of HCC in patients with MASLD.^22^^,^^23^

In this study, we investigated for the first time the association between GRS and MASLD-related HCC and cirrhosis in Latin Americans. We developed a GRS based on the presence of risk-increasing alleles in *PNPLA3*, *MBOAT7*, *TM6SF2*, and *HSD17B13*. In addition, we examined the correlation between GRS and immune modulation represented by cytokine levels in MASLD-related cirrhotic HCC and cirrhosis, with the goal of defining improved risk-assessment tools incorporating genetic and immune markers as well as helping understand the underlying mechanisms of HCC progression in this setting.

## Patients and methods

### Samples and study individuals

This study utilized data from the ESCALON network,^24^^,^^25^ a European–Latin American collaboration focused on evaluating clinical and genetic factors to identify biomarkers for the early diagnosis and treatment of hepatobiliary tumors (www.escalon.eu). Patient recruitment was based on availability rather than randomization, and information and blood samples were collected at each participating institution, recorded in a Research Electronic Data Capture (REDCap) registry.

This cohort included patients from Latin American countries, specifically Argentina, Brazil, Chile, Colombia, Ecuador, and Peru. Medical records, along with confirmatory imaging, pathology, and laboratory tests, were used to determine the etiology, tumor stage, and fibrosis stage. Detailed information on etiology, tumor stage, and other relevant data can be found in Table 1. HCC diagnoses were made based on biopsy or imaging criteria established by the American Association for the Study of Liver Diseases.^26^ For patients with cirrhotic HCC, the Barcelona Clinic Liver Cancer (BCLC) staging system was applied.^27^ The presence of severe fibrosis or cirrhosis was determined by the managing hepatologists using pathology (METAVIR ≥F3–F4) or liver transient elastography studies (>12.0 kPa). Patients with ALD were classified based on persistent steatohepatitis attributable to prolonged ethanol intake, defined as 30 g/day for women and 40 g/day for men, over a 10-year period. The diagnosis of MASLD was made by the managing hepatologist or through evidence of hepatic steatosis on pathology or imaging in the absence of other clear causes for hepatic steatosis. Individuals without viral hepatitis, MASLD, or ALD were categorized as having ‘other’ etiology, which included both known and unknown causes. Patients with a mixed etiology of liver disease, defined as any combination of HBV, HCV, MASLD, and ALD, were also categorized as ‘other’ etiology and excluded from the subgroup analysis. Only patients with sufficient data on liver disease etiology, tumor size, and fibrosis status were included. Exclusion criteria were HCC recurrence, non-HCC liver metastases, mixed-type HCC, age <18 years, and the presence of co-existing non-HCC malignancies. Informed written consent was obtained from each patient included in the study, and ethical approval was granted by the local and/or regional Ethics Committees of all centers. The study adheres to the ethical guidelines of the 1975 Declaration of Helsinki, as reflected in prior approval by the local and/or regional Ethics Committees of all participating centers.

**Table 1:** Clinical parameters for MASLD-related liver disease.

Variable	HCC (n = 142)	Cirrhosis (n = 242)	NCLD (n = 113)
Age, median (IQR)	69 (63–74)	64 (58–69)	60 (50–65)
Male, n (%)	80 (56.3)	97 (40.1)	47 (78.3)
Cirrhosis, n (%)	133 (93.7)	242 (100)	0
Ethnicity, n (%)			
Europeans	4 (2.8)	22 (9.1)	10 (8.8)
Latin Americans	136 (95.8)	219 (90.5)	102 (90.3)
Others	2 (1.4)	1 (0.4)	1 (0.9)
BMI, median (IQR)	28.1 (25.0–32.6)	28.7 (26.0–32.0)	27.3 (24.6-31.1)
Diabetes, n (%)	84 (59.2)	118 (48.8)	29 (25.7)
Stage, n (%)∗			
0–A	79 (55.6)	NA	NA
B	29 (20.4)	NA	NA
C–D	24 (16.9)	NA	NA
Unknown	1 (0.7)	NA	NA
*PNPLA3* rs738409			
CC	8 (5.6)	25 (10.3)	33 (29.2)
CG	37 (26.1)	90 (37.2)	50 (44.2)
GG	97 (68.3)	127 (52.5)	30 (26.5)
*MBOAT7* rs641738			
CC	52 (36.6)	79 (32.6)	44 (38.9)
CT	61 (43.0)	122 (50.4)	60 (53.1)
TT	29 (20.4)	41 (16.9)	9 (8.0)
*HSD17B13* rs72613567			
TT	126 (88.7)	196 (81.0)	83 (73.4)
TAT	15 (10.6)	43 (17.8)	24 (21.2)
TATA	1 (0.7)	3 (1.2)	6 (5.3)
*TM6SF2* rs58542926			
CC	126 (88.7)	218 (90.1)	101 (89.4)
CT	16 (11.3)	24 (9.9)	12 (10.6)
TT	0	0	0

∗ Only patients with cirrhotic HCC were assessed using the BCLC stage. ALD, alcoholic liver disease; HCC, hepatocellular carcinoma; HSD17B13, hydroxysteroid 17-beta dehydrogenase 13; MASLD, metabolic dysfunction-associated steatotic liver disease; MBOAT7, membrane-bound O-acyltransferase domain-containing protein 7; NA, not available; NCLD, non-cirrhotic liver disease; PNPLA3, patatin-like phospholipase domain-containing protein 3; TM6SF2, transmembrane 6 superfamily member 2.

### Sample collection

Serum samples were prospectively collected starting in 2019 for HCC biomarker discovery and validation studies. A data monitor regularly reviewed all data. The control group was required to have a minimum follow-up of 24 months after biomarker assessment to confirm the absence of HCC. Serum samples from patients diagnosed with HCC were collected at the time of diagnosis. DNA was isolated from the peripheral blood of 983 patients from Argentina, Peru, Chile, Colombia, Brazil, and Ecuador.

### Genotyping

For SNPs, 20 ng of genomic DNA was used in the qPCR reaction mix. The SNPs of *PNPLA3* rs738409 (C>G) (ThermoFisher, Assay ID C______7241_10), *MBOAT7* rs641738 (C>T) (ThermoFisher, Waltham, MA, USA, Assay ID C___8716820_10), *HSD17B13* rs72613567 (T>TA) (ThermoFisher, Assay ID AN7D39Z), and *TM6SF2* rs58542926 (C>T) (ThermoFisher, Assay ID C__89463510_10) were genotyped using TaqMan pre-designed SNP genotyping assays (ThermoFisher). Genotyping was performed on the StepOnePlus Real-Time PCR System (ThermoFisher) with a Custom TaqMan SNP Genotyping Assay (Applied Biosystems, Foster City, CA, USA). The qPCR reactions were carried out in a 10-μl volume containing 4 μl of genomic DNA (5 ng/μl) and 6 μl of Genotyping Master Mix with probe.

### Genetic risk score

*PNPLA3* rs738409 (C>G), *MBOAT7* rs641738 (C>T), *HSD17B13* rs72613567 (T>TA), and *TM6SF2* rs58542926 (C>T) were coded as 0, 1, and 2 for non-carriers, heterozygous carriers, and homozygous carriers of the risk-increasing allele, respectively. For *PNPLA3* rs738409 (C>G), *MBOAT7* rs641738 (C>T), and *TM6SF2* rs58542926 (C>T), the risk-increasing allele was the minor allele (G for *PNPLA3*, and T for *MBOAT7* and *TM6SF2*). In contrast, for *HSD17B13* rs72613567 (T>TA), the T allele was considered the risk allele, as the minor TA allele has been previously associated with protection from chronic liver disease.^17^^,^^28^ For the calculation of odds ratios (ORs) in these participants, a combined GRS was calculated as the sum of the risk-increasing alleles (range, 0–8). Because of the small number of individuals with scores of 0, 1, 7, and 8, those with scores of 0, 1, and 2 were grouped together, and those with scores of 6, 7, and 8 were combined into another group. For comparing cytokine levels between GRS groups, patients with a GRS of 0–4 were classified as the low group, and those with a GRS of 5–8 were classified as the high group.

### Measurement of immune markers

Circulating cytokines were measured using the Bio-Plex platform with the Bio-Plex Human Cytokine 40-Plex panel and Single-Plex kits for Pentraxin-3, MMP-2, and MMP-3 (Bio-Rad, Hercules, CA, USA). A total of 43 analytes were tested following the manufacturer's protocol. Serum samples (20 μl) were diluted 4 × with sample diluent (60 μl). Anti-cytokine conjugated beads (55 μl) were plated in a 96-well plate, washed twice, and incubated with 50 μl of cytokine standards, kit controls, or serum samples for 60 min. The plates were washed three times with Bio-Plex wash buffer, then 25 μl of detection antibody was added and incubated for 30 min. After further washes, 50 μl of streptavidin–phycoerythrin was added, and incubation was performed for 10 min before washing and suspending the beads in Bio-Plex assay buffer. Data were acquired using a Bio-Plex 200 system (Bio-Rad), with analysis conducted on Bio-Plex Manager 6.2 software. Standard curves were calculated using five-parameter logistic regression with automated weighting. The highest and lowest reliable values of the standard curve were used to determine the limits of quantification. To ensure reproducibility, serum from healthy controls and patients with HCV were pooled, aliquoted, and included at one position of each plate. Additionally, a technical replicate was performed for the Cytokine 40-Plex panel. The coefficient of variation (%CV) was calculated as SD/mean, cytokines with a coefficient of variation over 0.3 (30%), including IL-10, CCL19, CCL7, IL-6, CXCL5, CCL20, GM-CSF, CCL26, IL-4, CXCL13, IL-16, CCL17, and CCL21 were excluded. Additionally, two cytokines with values near the lower limit of detection for most samples, IL-2 and CX3CL1, were also excluded.

### Statistical analysis

Statistical analyses were conducted using SPSS (version 28.0.1.0, IBM; Armonk, NY, USA), RStudio (version 4.5.0, RStudio, Inc. Boston, MA, USA), and GraphPad Prism (version 8.0.2, GraphPad Software, San Diego, CA, USA). Continuous variables were reported as medians with IQR, while categorical variables were expressed as percentages. Descriptive statistics summarized patient characteristics for the case and control groups. The Χ^2^ test and Fisher’s exact test were applied to analyze dichotomous variables, and the Mann–Whitney *U* test was used for continuous variables. Binary logistic regression was used to examine the ORs between the four selected SNPs or GRS and liver diseases. Spearman correlation was used to evaluate the relationship between GRS and cytokine levels, while multivariate linear regression further assessed their association after adjusting for clinical characteristics. The AUC was calculated to assess the performance of *PNPLA3* and the GRS, with Delong’s test applied to compare AUCs. Power calculations were performed using PS software (https://cqsclinical.app.vumc.org/ps/). Statistical significance was defined as a two-tailed *p* value <0.05.

## Results

### Baseline characteristics of participants

A total of 972 participants were included in this study (Table S1). The cohort comprised 267 patients with HCC (91% cirrhotic), 455 patients with cirrhosis, 139 individuals with non-cirrhotic liver diseases (NCLD), and 111 healthy controls. The overall median age was 64 years (IQR: 57–70). Among HCC cases, the median age was 68 years (IQR: 62–73), and 66% were male. MASLD was the primary etiology of HCC (53%), cirrhosis (53%), and NCLD (81%).

### Risk assessment of the four selected SNPs for MASLD-related HCC and cirrhosis

**Fig. 1:**
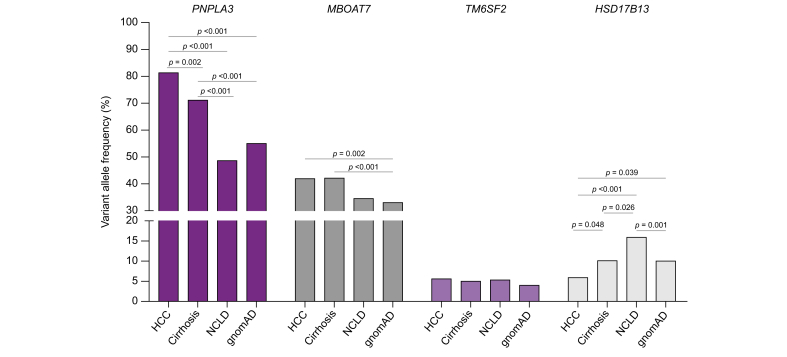
Variant allele frequencies of the four SNPs in MASLD-related liver disease compared with those reported in the gnomAD database. The variant allele frequencies of *PNPLA3* rs738409, *MBOAT7* rs641738, *TM6SF2* rs58542926, and *HSD17B13* rs72613567 in patients with MASLD-related HCC, cirrhosis, and non-cirrhotic liver disease (NCLD) were compared with each other and those in the Latin American population of the gnomAD database. The Χ^2^ test was used to calculate the *p* values. HCC, hepatocellular carcinoma; MASLD, metabolic dysfunction-associated steatotic liver disease.

We first calculated the variant allele frequency (VAF) of the four SNPs in patients with MASLD-related HCC and cirrhosis and compared them with the Hispanic population in the gnomAD database, a public genome database which contains genomic data on 141,456 individuals including 15,724 Hispanics (https://gnomad.broadinstitute.org/, Fig. 1). The VAFs in the gnomAD database were 55%, 33%, 4%, and 10% for *PNPLA3* rs738409, *MBOAT7* rs641738, *TM6SF2* rs58542926, and *HSD17B13* rs72613567, respectively. The VAFs of *PNPLA3* (81.3%, *p* <0.05), *MBOAT7* (41.9%), and *TM6SF2* (5.6%) were higher in patients with HCC, whereas the VAF of *HSD17B13* (5.9%, *p* <0.05) was lower in the HCC group compared with the gnomAD database.

### PNPLA3 is associated with the development of MASLD-related HCC

We next assessed the effects of individual SNPs in MASLD-related liver disease. As shown in Table 1, the GG risk genotype of *PNPLA3* was more prevalent in HCC cases compared with cirrhosis (68.3% *vs.* 52.5%, *p* = 0.002). Similarly, the TT risk allele of *HSD17B13* (88.7% *vs.* 81.0%, *p* = 0.047) was higher in HCC compared with cirrhosis. The percentage of the TT genotype for *MBOAT7* (20.4% *vs.* 16.9%, *p* = 0.394) was higher in HCC compared with cirrhosis, although no statistical significance was found. The ratio of CT-*TM6SF2* was similar between the HCC and cirrhosis groups, and no TT-*TM6SF2* genotype was observed in either group.

To reduce potential bias, we adjusted the OR by age, sex, BMI, and ethnicity. Patients with cirrhosis (n = 242) and the *PNPLA3*-GG genotype showed a 2.8-fold increase in the odds of developing cirrhotic HCC compared with those with the CC genotype (OR: 2.805, 95% CI 1.083–7.264, *p* = 0.034, Table 2). No statistical significance was observed for the OR of SNPs in *MBOAT7*, *HSD17B13*, and *TM6SF2* in MASLD-related cirrhotic HCC compared with cirrhosis. When compared with NCLD (Table 2), the odds of developing HCC increased with the presence of *PNPLA3* CG and GG genotypes, with ORs of 4.1 and 13.8, respectively. Additionally, carriers of the *HSD17B1*3 TATA genotype demonstrated a protective effect, with an OR of 0.024. Both associations met the power analysis threshold (Table S2), indicating a potential role for *PNPLA3* and *HSD17B13* in MASLD-related HCC development. When further adjusting the OR for diabetes (Table S3), no statistically significant association was observed for the *PNPLA3* GG genotype in the comparison between cirrhotic HCC and cirrhosis. Next, subgroup analyses based on different etiologies were performed, except for the heterozygous TA genotype of *HSD17B13*, which showed a protective role in ALD-related HCC development (OR: 0.136, 95% CI 0.034–0.536, *p* = 0.004), no other SNPs were found to be associated with viral- or ALD-related HCC development (Table S4).

**Table 2:** The impact of the four individual SNPs in MASLD-related liver disease.

SNPs	Cirrhotic HCC *vs.* cirrhosis		Cirrhotic HCC *vs.* NCLD		Cirrhosis *vs.* NCLD	
	OR 95% CI (adjusted)	*p* value	OR 95% CI (adjusted)	*p* value	OR 95% CI (adjusted)	*p* value
*PNPLA3*						
CC	Reference	NA	Reference	NA	Reference	NA
CG	1.498 (0.574–3.907)	0.409	4.118 (1.318–12.87)	0.015	3.208 (1.560–6.597)	0.002
GG	2.805 (1.083–7.264)	0.034	13.81 (4.534–42.08)	<0.001	6.873 (3.293–14.35)	<0.001
*MBOAT7*						
CC	Reference	NA	Reference	NA	Reference	NA
CT	0.683 (0.408–1.142)	0.146	0.624 (0.309–1.257)	0.187	1.075 (0.637–1.813)	0.788
TT	1.094 (0.582–2.058)	0.780	2.557 (0.872–7.500)	0.087	2.238 (0.909–5.513)	0.080
*HSD17B13*						
TT	Reference	NA	Reference	NA	Reference	NA
TAT	0.595 (0.302–1.172)	0.133	0.358 (0.141–0.908)	0.031	0.656 (0.357–1.206)	0.175
TATA	0.333 (0.024–4.699)	0.415	0.024 (0.002–0.370)	0.008	0.112 (0.023–0.551)	0.007
*TM6SF2*						
CC	Reference	NA	Reference	NA	Reference	NA
CT	1.397 (0.658–2.962)	0.384	0.859 (0.326–2.261)	0.758	0.828 (0.381–1.796)	0.632
TT	NA	NA	NA	NA	NA	NA

The odds ratio (OR) was adjusted for age, sex, BMI, and ethnicity. HSD17B13, hydroxysteroid 17-beta dehydrogenase 13; MASLD, metabolic dysfunction-associated steatotic liver disease; MBOAT7, membrane-bound O-acyltransferase domain-containing protein 7; NA, not available; NCLD, non-cirrhotic liver disease; OR, odds ratio; PNPLA3, patatin-like phospholipase domain-containing protein 3; TM6SF2, transmembrane 6 superfamily member 2.

### PNPLA3 and HSD17B13 are associated with the development of MASLD-related cirrhosis

We compared the four SNPs between the MASLD-related cirrhosis (n = 242) and NCLD (n = 113) groups (Table 1). The percentage of GG-*PNPLA3* (52.5% *vs.* 26.5%, *p* <0.001) and TT-*MBOAT7* (16.9% *vs.* 8.0%, *p* = 0.024) was higher in the cirrhosis group compared with the NCLD group. Similarly, the percentage of TT-*HSD17B13* (81.0% *vs.* 73.4%, *p* = 0.107) was higher in the cirrhosis group, although the difference was not statistically significant. The percentage of CT-*TM6SF2* was similar between the cirrhosis and NCLD groups.

As shown in Table 2, compared with NCLD, when adjusted for sex, age, BMI, and ethnicity, patients with the CG-*PNPLA3* genotype exhibited a 3.2-fold increase in the odds of developing cirrhosis (OR: 3.208, 95% CI 1.560–6.597, *p* = 0.002). The odds further increased to 6.9-fold for patients with the GG-*PNPLA3* genotype (OR: 6.873, 95% CI 3.293–14.35, *p* <0.001). In contrast, the TA genotype of *HSD17B13* showed a protective role against cirrhosis development, with patients carrying the TATA-*HSD17B13* genotype having lower odds of developing cirrhosis (OR: 0.112, 95% CI 0.023–0.551, *p* = 0.007). Notably, the ORs for *PNPLA3* and *HSD17B13* also met the threshold established by the power analysis (Table S2). However, no statistical significance was observed between TT-*MBOAT7* (OR: 2.238, 95% CI 0.909–5.513, *p* = 0.080) or CT-*TM6SF2* (OR: 0.828, 95% CI 0.381–1.796, *p* = 0.632) and MASLD-related cirrhosis. When further adjusting the ORs for diabetes (Table S3), the associations for the *PNPLA3* GG genotype and the *HSD17B1*3 TATA genotype remained statistically significant. Next, we investigated the association in different etiological subgroups. The viral group did not show any associations between the four SNPs and cirrhosis development (Table S5), and there was an insufficient number of patients with ALD-related NCLD to be analyzed in this study. In summary, our findings suggest that SNPs in *PNPLA3* and *HSD17B13* are associated with MASLD-related cirrhosis development.

### GRS were associated with a higher risk of developing MASLD-related HCC and cirrhosis

**Fig. 2:**
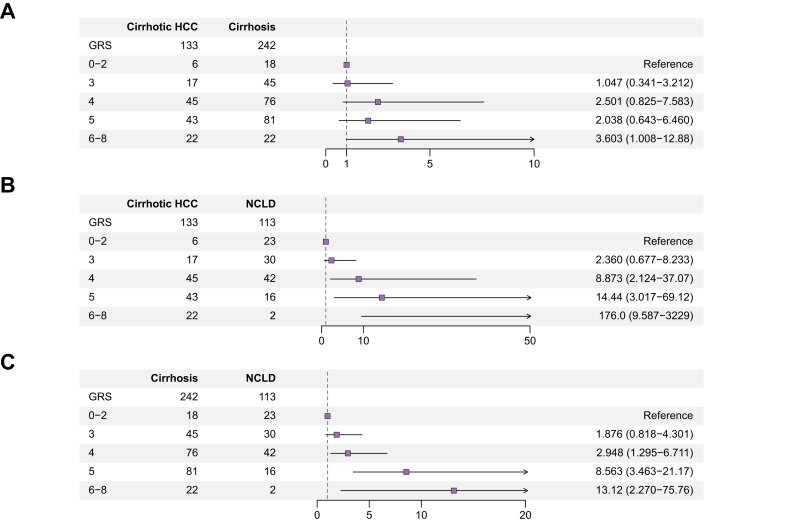
Performance of the GRS in stratifying high-risk populations for MASLD-related liver disease. Association of the genetic risk score (GRS) with the risk of cirrhotic HCC compared with cirrhosis (A) or NCLD (B), and cirrhosis compared with NCLD (C) in Latin American patients with MASLD. Odds ratios were calculated using binary logistic regression, adjusted for age, sex, BMI, and ethnicity. Error bars represent 95% CIs. HCC, hepatocellular carcinoma; MASLD, metabolic dysfunction-associated steatotic liver disease; NCLD, non-cirrhotic liver disease.

We assessed the collective impact of SNPs by calculating a GRS. After adjusting for age, sex, BMI, and ethnicity, individuals with a GRS of 6–8 exhibited 3.6-fold increased odds of developing HCC (OR: 3.603, 95% CI 1.008–12.88, *p* = 0.049) compared with those with a GRS of 0–2 when comparing cirrhotic HCC to cirrhosis in MASLD (Fig. 2A). In contrast, individuals with a GRS of 3–5 showed no statistically significant difference in cirrhotic HCC risk compared with those with a GRS of 0–2, suggesting that a higher GRS is associated with an increased risk of HCC development. When further adjusting the ORs for diabetes (Fig. S1A), no statistically significant association was observed for GRS 6–8 compared with GRS 0–2. Notably, power analysis (Table S2) indicated that the sample size was insufficient to detect an OR that small. When comparing cirrhotic HCC to NCLD (Fig. 2B), the OR increased in a stepwise trend with rising GRS values: 8.9-fold for GRS 4 (95% CI: 2.2–37.1), 14.4-fold for GRS 5 (95% CI: 3.0–69.1), and 176.0-fold for GRS 6–8 (95% CI: 9.6–3,229). Similarly, when comparing MASLD-related cirrhosis to NCLD (Fig. 2C), a stepwise increase in the odds was observed among patients with GRS values of 4 (OR: 2.948, 95% CI: 1.295–6.711, *p* = 0.010), 5 (OR: 8.563, 95% CI: 3.463–21.17, *p* <0.001), and 6–8 (OR: 13.12, 95% CI: 2.270–75.76, *p* = 0.004) compared with those with a GRS of 0–2. These associations remained statistically significant after adjustment for diabetes (Fig. S1B and C).

Compared with individual SNPs, although no statistically significant difference was observed in the AUC for the GRS and *PNPLA3* in MASLD-related HCC and cirrhosis development (Table S6), the OR for GRS 6–8 was substantially higher than that for *PNPLA3* (3.603 *vs.* 2.805 or 176.0 *vs.* 13.81 for cirrhotic HCC, and 13.12 *vs.* 6.873 for cirrhosis). Additionally, the ORs for GRS 6–8 when comparing cirrhotic HCC or cirrhosis with NCLD met the threshold established by the power analysis (Table S2), suggesting a synergistic role of the four SNPs in MASLD-related HCC and cirrhosis development.

### Patients with MASLD-related cirrhotic HCC with lower GRS exhibited higher cytokine serum levels compared with those with higher GRS

To further investigate the potential association of inflammation and HCC, as well as genetic-immune combined risk stratification, we classified a subgroup of patients into low- and high-GRS groups (Table 3), defined as GRS 0-4 (n = 56) and GRS 5-8 (n = 51), respectively. We then measured the levels of 43 cytokines previously reported to be associated with the progression of MASLD-related liver disease.^29^^,^^30^ Age, sex, lean or overweight status, BMI, and BCLC stage were comparable between the GRS 0–4 and GRS 5–8 groups in cirrhotic HCC.

**Table 3:** Clinical parameters for MASLD-related liver disease with GRS 0-4 and GRS 5-8.

	Cirrhotic HCC			Cirrhosis		
	GRS 0–4	GRS 5–8	*p* value	GRS 0–4	GRS 5–8	*p* value
N	56	51	–	69	42	–
Age, median (IQR)	69 (63–74)	69 (65–75)	0.798	66 (62–71)	63 (57–69)	0.111
Male, n (%)	27 (48.2)	30 (58.8)	0.272	25 (36.2)	16 (38.1)	0.844
Cirrhosis, n (%)	56 (100)	51 (100)	–	69 (100)	42 (100)	–
BMI, median (IQR)	27.0 (24.6–32.4)	28.0 (25.0–33.0)	0.868	27.0 (24.2–31.2)	27.1 (24.0–30.0)	0.992
Lean (BMI <25), n (%)	15 (26.8)	12 (23.5)	0.699	22 (31.9)	13 (31.0)	0.918
Overweight (BMI ≥25), n (%)	41 (73.2)	39 (76.5)	0.699	47 (68.1)	29 (69.0)	0.918
BCLC stage, n (%)						
0–A	33 (58.9)	33 (64.7)	0.539	NA	NA	NA
B	14 (25.0)	10 (19.6)	0.504	NA	NA	NA
C–D	9 (16.1)	8 (15.7)	0.957	NA	NA	NA

BCLC, Barcelona Clinic Liver Cancer Staging System; GRS, genetic risk score; HCC, hepatocellular carcinoma; HSD17B13, hydroxysteroid 17-beta dehydrogenase 13; MBOAT7, membrane-bound O-acyltransferase domain-containing protein 7; NA, not available; PNPLA3, patatin-like phospholipase domain-containing protein 3; TM6SF2, transmembrane 6 superfamily member 2.

**Fig. 3:**
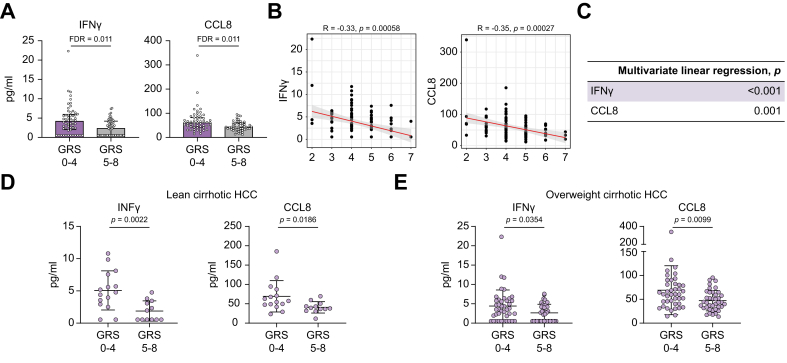
Cytokine levels in MASLD-related cirrhotic HCC stratified by genetic risk score (GRS). (A) In the overall MASLD-related cirrhotic HCC group, IFNγ and CCL8 showed higher levels with FDR <0.05 in patients with GRS 0–4 (n = 56) compared with those with GRS 5–8 (n = 51). (B) IFNγ and CCL8 exhibited decreased levels with increasing GRS in overall MASLD-related cirrhotic HCC, as determined by Spearman correlation, and the *p* values were further adjusted for age, sex, and BMI using multivariate linear regression (C). (D) In the lean MASLD-related cirrhotic HCC group, IFNγ and CCL8 displayed higher levels in GRS 0–4 (n = 15) compared with GRS 5–8 (n = 12). (E) In the overweight MASLD-related cirrhotic HCC group, IFNγ and CCL8 also showed higher levels in GRS 0–4 (n = 41) compared with GRS 5–8 (n = 39). For plots A, D, and E, *p* values were calculated using the Mann–Whitney *U* test. FDR, false discovery rate; HCC, hepatocellular carcinoma; IFNγ, interferon-gamma; MASLD, metabolic dysfunction-associated steatotic liver disease.

As shown in Table S7, the manufacturer-provided kit control exhibited consistent performance across the plates, with only one cytokine showing variability. Fifteen cytokines were excluded from further analysis because of either a lower limit of detection or a coefficient of variation >30%. Additionally, the overall analytical performance of the assay demonstrated low intra-assay and interassay coefficient of variation for key analytes, ensuring reliable results across replicate measurements. To compare the cytokine levels, we used both *p* values and adjusted *p* values (FDR) to assess statistical significance. Of the remaining 28 cytokines, two with an FDR <0.05 (Fig. 3A) and six with *p* <0.05 (Fig. S2A) exhibited increased levels in the low-GRS group compared with the high-GRS group, suggesting a noninflammatory pathway to HCC development in these patients (Table S8). These markers included interferon-gamma (IFNγ), CCL8 (FDR <0.05), CXCL9, tumor necrosis factor-alpha (TNFα), CCL13, CXCL10, CXCL16, and CCL2 (*p* <0.05). When stratified by early-stage and late-stage HCC, the trend remained consistent, with higher cytokine levels observed in the GRS 0–4 group compared with the GRS 5–8 group in both early and late HCC (Fig. S3). Next, we conducted Spearman correlation analysis to examine the association between individual GRS and cytokine levels for these cytokines. We observed IFNγ and CCL8 exhibited decreased levels as GRS increased (*p* <0.05, Fig. 3B), this was also observed for CXCL9, TNFα, CCL13, CXCL10, and CCL2 (*p* <0.05, Fig. S2B). A multivariate linear regression analysis, adjusted for age, sex, and BMI, confirmed that the levels of IFNγ, CCL8, CXCL9, TNFα, CCL13, CXCL10, and CCL2 was significantly higher in the GRS 0–4 group compared with the GRS 5–8 group in cirrhotic HCC (Fig. 3C and Fig. S2C). In summary, these findings suggest that higher GRS is associated with diminished immune activity in MASLD-related cirrhotic HCC.

Next, we investigated whether markers in lean or overweight patients with cirrhotic HCC were associated with GRS. Interestingly, patients with low GRS exhibited higher levels of CCL8 and IFNγ in both lean and overweight HCC (Fig. 3D and E) compared with those with high GRS. In contrast, CXCL16 and CCL2 were elevated exclusively in lean cirrhotic HCC with low GRS (Fig. S2D), whereas CXCL9 and CXCL10 were exclusively increased in overweight HCC with low GRS (Fig. S2E). It should be noted that the sample size for lean patients with HCC is small, with 12 patients in the high-GRS group and 15 in the low-GRS group. Nevertheless, these findings suggest that immune status may vary between lean and overweight patients with cirrhotic HCC with different GRS, potentially indicating distinct mechanisms of HCC development.

### Patients with MASLD-related cirrhosis with lower GRS exhibited lower cytokine serum levels compared with those with a higher GRS

**Fig. 4:**
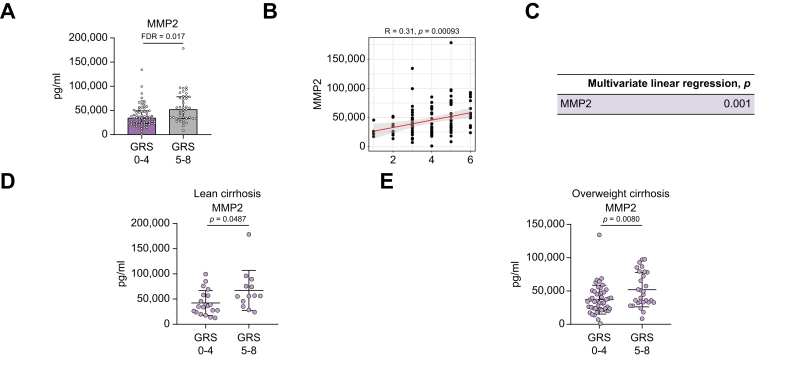
Cytokine levels in MASLD-related cirrhosis stratified by genetic risk score (GRS). (A) In the overall MASLD-related cirrhosis group, MMP2 exhibited higher levels in patients with GRS 5–8 (n = 42) compared with those with GRS 0–4 (n = 69). (B) Using Spearman correlation analysis, MMP2 also showed elevated levels with increasing GRS in the overall MASLD-related cirrhosis group, and the *p* values were further adjusted for age, sex, and BMI using multivariate linear regression (C). When stratified by lean and overweight cirrhosis, (D) MMP2 demonstrated increased levels in lean cirrhosis patients with GRS 5–8 (n = 13) compared with GRS 0–4 (n = 22). (E) In the overweight MASLD-related cirrhosis, MMP2 also showed increased levels in overweight patients with cirrhosis with GRS 5–8 (n = 29) compared with GRS 0–4 (n = 47). The Mann–Whitney *U* test was used to calculate *p* values for plots A, D, and E. MASLD, metabolic dysfunction-associated steatotic liver disease; MMP2, matrix metallopeptidase 2.

Compared with the GRS 0–4 group, patients with cirrhosis with GRS 5–8 exhibited increased levels of MMP2 (FDR <0.05, Fig. 4A), CCL1, and CXCL8 (*p* <0.05, Fig. S4A). Next, we assessed the association between individual GRS and cytokine levels for these three cytokines. Higher levels of MMP2 (Fig. 4B) and CXCL8 (Fig. S4B) were strongly associated with a higher GRS, but this was not observed for CCL1. After adjusting for age, sex, and BMI, MMP2 remained correlated with increased GRS (Fig. 4C), whereas this correlation was not found for CXCL8. When stratified into lean and overweight cirrhosis groups, patients with GRS 5–8 consistently showed higher levels of MMP2 in both groups compared with GRS 0–4 (Fig. 4D and E). However, CCL1 was only associated with lean cirrhosis in this comparison (Fig. S4C), whereas CXCL8 did not exhibit statistical significance in either the lean or overweight cirrhosis groups.

Therefore, in contrast with cirrhotic HCC, we found that a higher GRS is associated with increased levels of cytokines in patients with MASLD-related cirrhosis, suggesting differential immune-related HCC-development mechanisms in MASLD that can be used for improved risk stratification.

## Discussion

MASLD poses a significant global health burden, particularly in Latin America, where its prevalence is exceptionally high.^7^^,^^8^ In this study, we assessed, for the first time, the correlation of GRS in MASLD-related liver disease in Hispanics, finding a 13.1-fold and 3.6-fold higher risk of developing cirrhosis and HCC, respectively, with GRS 6–8. In addition, we found an accompanied differential immune regulation associated to GRS in those with MASLD-HCC compared with MASLD-cirrhosis.

Several studies have shown that patients with a high GRS, calculated using different combinations of SNPs, are more likely to develop HCC.^17^^,^^19^^,^^20^ One retrospective study found a 29-fold higher risk of HCC associated with a GRS consisting of *PNPLA3*, *TM6SF2*, and *HSD17B13*,^17^ which is significantly higher than the ORs calculated in our study (3.6-fold). It is also important to note that the controls in the referenced study were individuals without cirrhosis, whereas in our study, HCC cases were compared with both patients with cirrhosis and those with NCLD. Notably, the ORs increased substantially – by 176-fold – when compared with the NCLD group. However, despite statistical significance, the ORs of 176.0 for GRS 6–8 *vs.* 0–2 should be interpreted cautiously because of the extremely wide confidence interval (9.587–3,229), indicating substantial uncertainty likely driven by small subgroup sizes. Additionally, two prospective studies demonstrated that a high GRS could predict HCC development.^19^^,^^20^ However, these studies focused on European populations. The ratio of *PNPLA3* CG/GG (below 55%) in patients with cirrhosis in these studies^19^^,^^20^ is much lower than in our Latin American cohort, which may reflect differences in etiologies and ethnic backgrounds.^31^

Diabetes is an important risk factor for MASLD-related HCC. In our primary analysis, we did not adjust the ORs for diabetes, as it is a core diagnostic component of MASLD and may function as an intermediate variable in the causal pathway. Although the associations for GRS 6–8 and *PNPLA3* GG genotype in the comparison between cirrhotic HCC and cirrhosis did not reach statistical significance after adjusting for diabetes, the *p* values approached significance. To preserve a strict clinical interpretability and biological relevance of our findings, we focused on unadjusted diabetes data. Nevertheless, this variable should be considered in larger studies because of its potential impact.

A few studies have examined single SNPs and their association with liver disease in Latin America, including our previous research on *TLL1*,^32^ *STAT4*,^33^ and *MBOAT7*.^34^ However, except for *MBOAT7*, no association was found between the other SNPs and MASLD-related liver disease. A study from Brazil reported a higher ratio of *PNPLA3* CG/GG (70%) in patients with MASLD,^35^ whereas another Brazilian study showed a lower ratio of *PNPLA3* CG/GG (below 50%) in patients with HCV,^36^ further emphasizing the role of underlying etiologies. In this study, *PNPLA3* was associated with the development of both cirrhosis and HCC. However, the combined GRS, which includes *PNPLA3*, demonstrated substantially higher ORs than *PNPLA3* alone in identifying patients at high risk for HCC and cirrhosis. Although no statistically significant difference was observed in the AUC between the GRS and *PNPLA3*, the stepwise increase in ORs with higher GRS values suggests that aggregating multiple risk variants may better capture cumulative genetic risk in MASLD. However, further studies are needed to evaluate whether this approach improves clinical risk stratification.

To the best of our knowledge, this study is the first to assess the association between GRS and immune dysregulation in patients with MASLD-related HCC. This approach allowed us to better understand the relational impact of SNPs in inflammation and HCC, and to evaluate potential novel biomarker combinations. A previous study in a European population found decreased intrahepatic inflammation in MASLD-related HCC compared with MASLD, suggesting an alternative noninflammatory path to cancer in these groups.^37^ We found that the serum levels of 28% of the cytokines studied, including IFNγ, CXCL9, and CXCL10, were lower in patients with a high GRS, suggesting a role for germline mutations in modulating immune changes. Several studies have reported that low levels of cytokines such as IFNγ,^38^^,^^39^ CXCL9,^40^ and CXCL10^41^ are associated with worse immune infiltration and poor prognosis in patients with HCC. It has been reported that CXCL9 and CXCL10 could recruit CD8+ T cells, Th1 cells, and NK cells.^42^^,^^43^ Thus, it is possible that lower levels of these pro-inflammatory cytokines may impair the body’s ability to mount an effective immune response against tumor cells. Particularly interesting is our finding of an immune dissociation between lean and non-lean MASLD-related cirrhosis and HCC as we exposed genetic mutations as potentially important in explaining inflammation-mediated liver complications. However, it is important to note that the sample size for lean patients with MASLD was small, which warrants caution in drawing definitive conclusions. Further studies, addressing peripheral and intrahepatic levels of these immune markers in relation to MASLD-related HCC will be needed to better address this interaction. Moreover, larger studies will be needed to evaluate the usefulness of these biomarkers in determining MASLD-related liver complications.

GRS has been associated with cirrhosis development.^17^^,^^44^ In our study, patients with MASLD with a higher GRS exhibited increased risk of cirrhosis, showing a two-fold higher combined effect compared with *PNPLA3* alone, suggesting a potential role of GRS in stratifying high-risk patients for MASLD-related cirrhosis development.

In contrast to the immune changes influenced by GRS in HCC, patients with MASLD with a high GRS exhibited increased peripheral cytokine levels, which may be linked to inflammation leading to cirrhosis development. Indeed, several studies have reported increased immune activity in the absence of HCC. *In vitro*, SNPs in *PNPLA3* were associated with increased cytokine levels, including IL-6^22^ and IL-8.^23^ *In vivo*, one study found no association between the *PNPLA3* variant and cytokine levels in MASLD-related liver disease,^45^ whereas another study reported that in ALD-related cirrhosis, *PNPLA3* SNPs were linked to increased levels of IL-8 and CXCL1.^23^ For *HSD17B13*, patients with liver disease carrying the risk allele TT exhibited elevated levels of IL-6 in blood.^46^ These findings strongly suggest an association between SNPs in lipid metabolism-related genes and increased immune activity in the absence of HCC, indicating distinct immune mechanisms underlying the relationship between GRS and immune profile in MASLD-related HCC and cirrhosis. We also investigated cytokine changes based on statistically significant SNPs, such as *PNPLA3* and *HSD17B13*. *PNPLA3* (CG/GG *vs.* CC) showed differences in two cytokine levels, whereas *HSD17B13* (TT *vs.* TAT/TATA) showed a difference in one cytokine level between high- and low-GRS groups (data not shown). As these SNPs may synergistically influence the severity of MASLD-related liver disease, a combined genetic-immune approach could offer valuable insights into the pathways leading to MASLD-related HCC and cirrhosis, as well as disease severity.

As we observed distinct cytokine pattern changes in MASLD-related HCC and cirrhosis, tracking cytokine alterations in these patients could help identify those at the highest risk of progressing from cirrhosis to HCC. Specifically, in our study, the presence of HCC in the high-GRS group was associated with decreased cytokine levels, suggesting that among patients with cirrhosis with a high GRS, those exhibiting decreased cytokine levels might be considered at higher risk for progression to HCC. Monitoring these changes could serve as an early indicator of progression, enabling more targeted interventions for high-risk individuals. Additionally, poor immune activity, as indicated by lower cytokine levels in patients with HCC, may help predict HCC prognosis. Specifically, immune markers such as IFNγ, CXCL9, and CXCL10 could serve as potential biomarkers in this regard. However, we did not assess the performance of cytokines for early HCC detection in this study because of the relatively small number of patients with early-stage HCC. Future studies with larger sample sizes are needed to further explore the role of these cytokines in early HCC detection and prognosis.

Our study has several limitations. Some subgroup analyses, especially those involving HBV, HCV, and ALD, did not reach the required statistical power threshold because of small sample sizes. As a result, these findings should be interpreted with caution owing to potential limitations in statistical reliability; however, we made a deliberate effort to collect samples from a unique and underrepresented region. Furthermore, our analysis was restricted to individuals from the Latin American general population, which may limit the generalizability of our findings to other ethnicities. However, this is also a strength as no study has assessed GRS in this manner in this population before, and when possible, we compared our SNP findings with the Hispanic cohort within the large gnomAD database. Our assessment of immune regulators is based on cytokine levels in serum, and the dynamic levels of cytokines can be influenced by external factors, such as diet, infection, or medication. This variability has historically clouded the ability to use cytokines as immune markers for assessing the severity of MASLD-related liver disease. Despite these limitations, our study provides important insights into specific implications of GRS in Hispanic populations as well as the potential association between GRS and immune markers in MASLD-related liver disease.

In conclusion, we found that GRS is associated with both the severity and immune alterations of MASLD-related liver disease in Hispanic populations.

## Abbreviations

ALD, alcohol-related liver disease; BCLC, Barcelona Clinic Liver Cancer staging system; FDR, false discovery rate; GRS, genetic risk score; HCC, hepatocellular carcinoma; HSD17B13, hydroxysteroid 17-beta dehydrogenase 13; IFNγ, interferon-gamma; MASH, metabolic dysfunction-associated steatohepatitis; MASLD, metabolic dysfunction-associated steatotic liver disease; MBOAT7, membrane-bound O-acyltransferase domain-containing protein 7; MMP2, matrix metallopeptidase 2; NAFLD, non-alcoholic fatty liver disease; NCLD, non-cirrhotic liver disease; OR, odds ratio; PNPLA3, patatin-like phospholipase domain-containing protein 3; SNPs, single-nucleotide polymorphisms; TM6SF2, transmembrane 6 superfamily member 2; TNFα, tumor necrosis factor-alpha; VAF, variant allele frequency.

## Financial support

This study was supported by the 10.13039/501100015383Foundation for Liver and Gastrointestinal Research (10.13039/501100015383SLO), the European–Latin American ESCALON consortium funded by the EU 10.13039/501100007601Horizon 2020 program, project number 825510, NIH-R21TW012390-01A1, FONDECYT 1241450, and NIH-R37CA297814-01.

## Authors’ contributions

Study concept and design: JD, AB. Performed experiments: SF, AG. Analyzed and interpreted the data: SF, BH. Collected clinical samples and data: DB, AM, EC, JDF, JP, LH, JMB, MA. Wrote the manuscript with the revision: SF, JD, AB. Supervised the project: JD, AB.

## Data availability

The data supporting the findings of this study are available from the corresponding author upon reasonable request.

## Conflicts of interest

The authors declare no conflicts of interest.

Please refer to the accompanying ICMJE disclosure forms for further details.
