# Supplementary material for: Genetic risk score correlates with immune profile and risk of HCC and cirrhosis development in Hispanics with MASLD
Source: JHEP Rep. 2025 Jul 2;7(10):101508. doi: 10.1016/j.jhepr.2025.101508 (PMC12455133; doi:10.1016/j.jhepr.2025.101508)
Supplement: Multimedia component 1 [file mmc1.pdf]

Genetic risk score correlates with immune profile and risk of HCC and cirrhosis development in Hispanics with MASLD

Siyu Fu, Anthonie Groothuismink, Domingo Balderramo, Angelo Z Mattos, Lisia Hoppe, Enrique Carrera, Javier Diaz-Ferrer, Jhon Prieto, Jesus M. Banales, Marco Arrese, Bettina E Hansen, Andre Boonstra, José D Debes

Table of contents

Fig. S1……………………………………………………………………….........................2

Fig. S2……………………………………………………………………….........................3

Fig. S3……………………………………………………………………….........................4

Fig. S4……………………………………………………………………….........................5

Table. S1…………………………...…………………………………………........................6

Table. S2…………………………...…………………………………………........................8

Table. S3…………………………...…………………………………………........................9

Table. S4…………………………...…………………………………………......................10

Table. S5…………………………...…………………………………………......................11

Table. S6…………………………...…………………………………………......................12

Table. S7…………………………...…………………………………………......................13

Table. S8…………………………...…………………………………………......................15

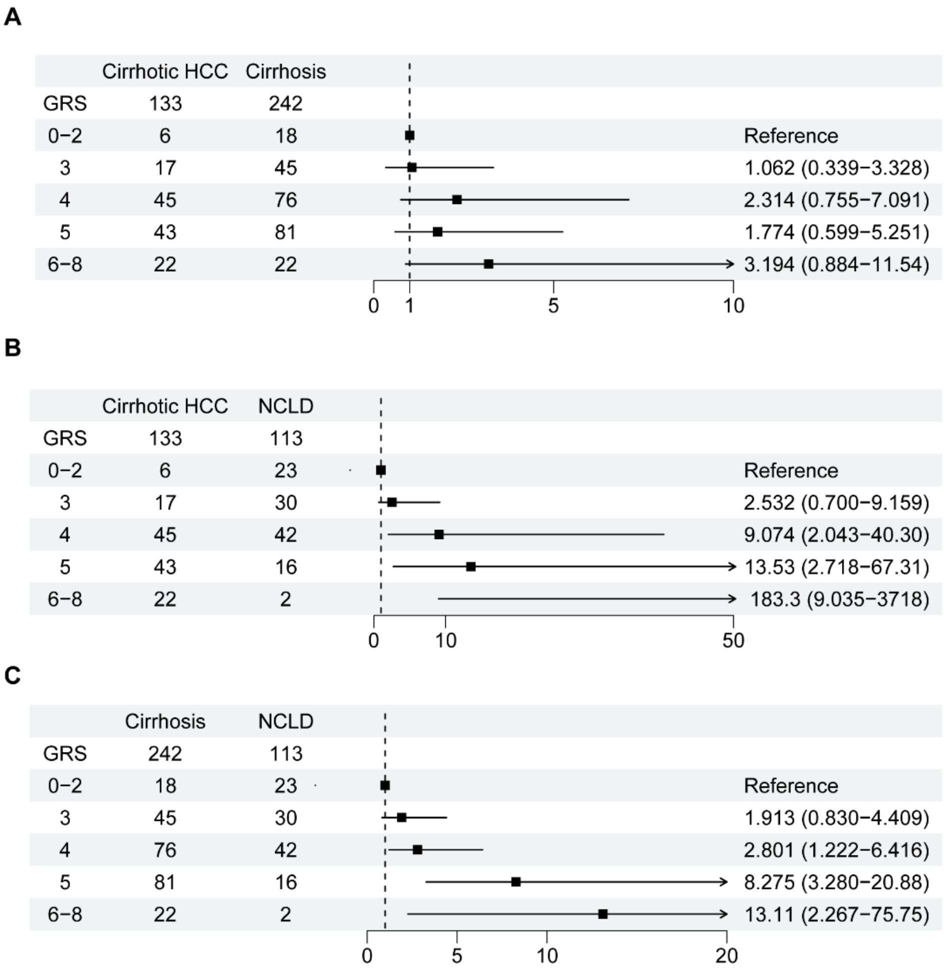

Fig. S1. Performance of the GRS in stratifying high-risk populations for MASLD-related liver disease. Association of the GRS with the risk of cirrhotic HCC compared to cirrhosis (A) or NCLD (B), and cirrhosis compared to NCLD (C) in Latin American patients with MASLD. Odds ratios were calculated using binary logistical regression, adjusted by age, gender, BMI, diabetes and ethnicity. Error bars represent 95% confidence intervals.

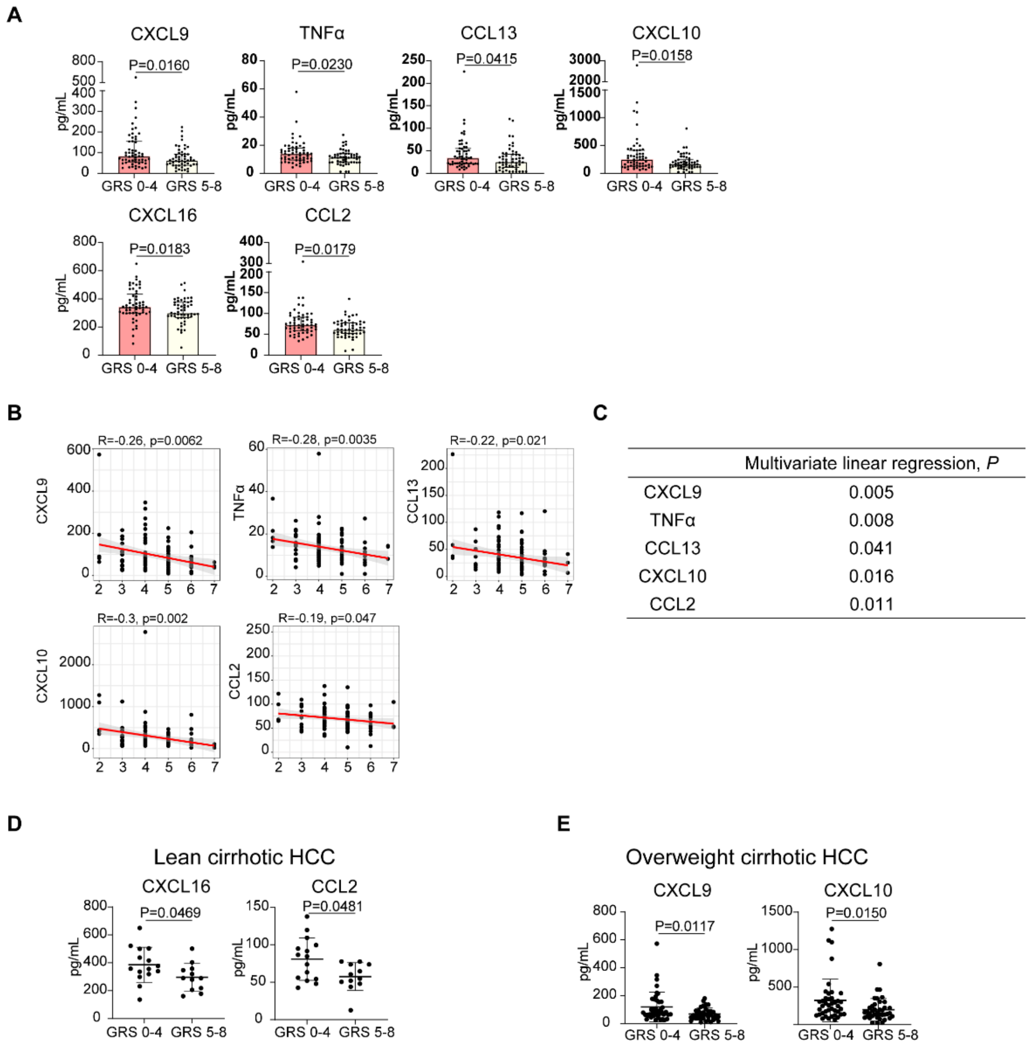

	Multivariate linear regression, *P*
CXCL9	0.005
TNFα	0.008
CCL13	0.041
CXCL10	0.016
CCL2	0.011

Fig. S2. Statistically significant cytokine levels (p<0.05) in MASLD-related cirrhotic HCC, categorized by high and low GRS. (A) 6 cytokines showed higher levels with p-value<0.05 in patients with GRS 0-4 (n=56) compared to those with GRS 5-8 (n=51). (B) 5 of 6 cytokines showed decreased levels with increasing GRS in MASLD-related cirrhotic HCC, as determined by Spearman correlation, and the p-values were further adjusted by age, gender, and BMI using multivariate linear regression (C). (D) In the lean MASLD-related cirrhotic HCC group, CXCL16 and CCL2 displayed increased levels in GRS 0-4 (n=15) compared to GRS 5-8 (n=12). (E) In the overweight MASLD-related cirrhotic HCC group, CXCL9 and CXCL10 exhibited higher levels in GRS 0-4 (n=41) compared to GRS 5-8 (n=39). For plots A, D, and E, p-values were calculated using Mann-Whitney U test.

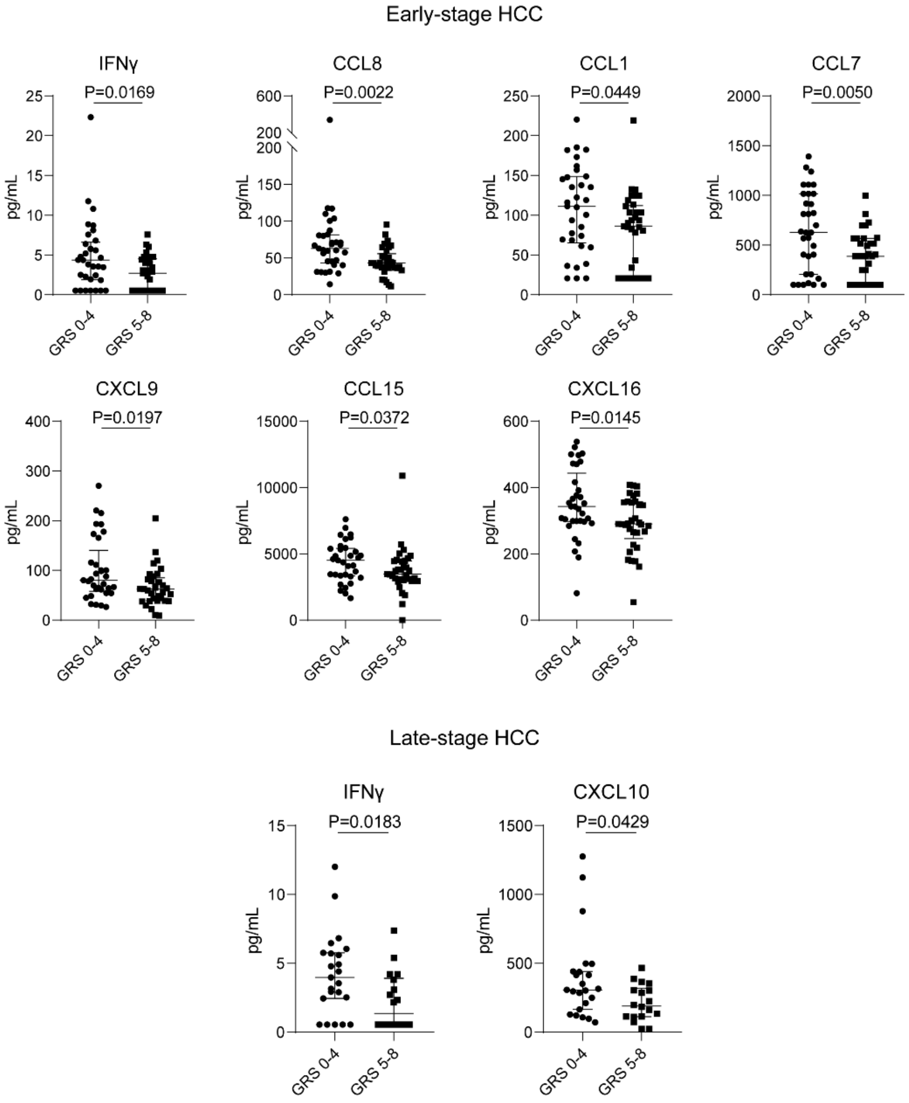

Fig. S3. Statistically significant cytokines levels in MASLD-related cirrhotic HCC, categorized by early and late stages. In early-stage HCC, 7 cytokines exhibited higher levels in GRS 0-4 (n=33) compared to GRS 5-8 (n=33). In late-stage HCC, 2 cytokines showed higher levels in the GRS 0-4 (n=23) compared to the GRS 5-8 (n=18). P-values were calculated using the Mann-Whitney U test.

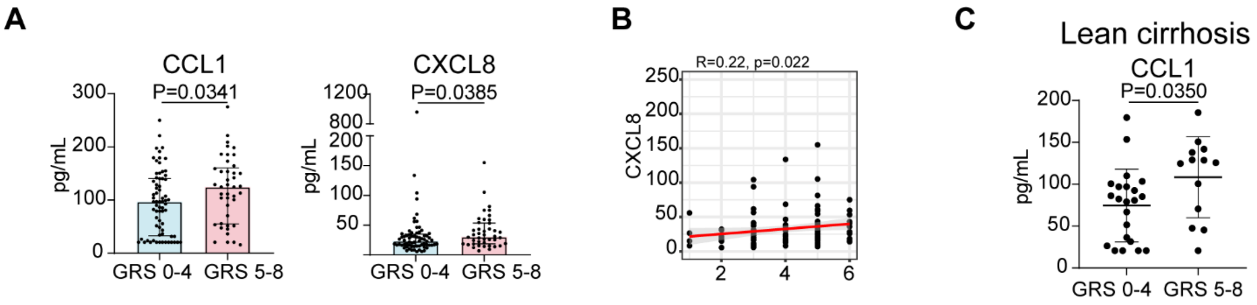

Fig. S4. Statistically significant cytokine levels ($p<0.05$) in MASLD-related cirrhosis, categorized by low and high GRS. (A) In the MASLD-related cirrhosis group, CCL1 and CXCL8 exhibited higher levels in patients with GRS 5-8 (n=42) compared to those with GRS 0-4 (n=69). (B) Using Spearman correlation analysis, CXCL8 also showed higher levels with increasing GRS in the overall MASLD-related cirrhosis. (C) CCL1 showed increased levels in lean cirrhosis patients with GRS 5-8 (n=13) compared to GRS 0-4 (n=22). The Mann-Whitney U test was used to calculate p-values for plots A and C.

Table S1. Clinical Characteristics of Latin American participants

Variable	HCC (n=267)	Cirrhosis (n=455)	NCLD (n=139)	Healthy controls (n=111)
Age, median (IQR)	68 (62-73)	63 (58-69)	59 (50-65)	58 (43-69)
Male, n (%)	175 (65.5%)	217 (47.7%)	59 (42.4%)	23 (20.7%)
Cirrhosis, n (%)	243 (91.0%)	455 (100%)	NA	NA
Ethnicity, n (%)				
Europeans	19 (7.1%)	76 (16.7%)	14 (10.1%)	32 (28.8%)
Americans	247 (93.9%)	375 (82.4%)	123 (88.5%)	78 (70.3%)
Others	1 (0.4%)	4 (0.9%)	2 (1.4%)	1 (0.9%)
BMI, median (IQR)	27.4 (24.2-31.0)	27.3 (24.4-30.8)	26.5 (24.5-29.7)	NA
Etiology, n (%)				
HBV	10 (3.7%)	7 (1.5%)	17 (12.2%)	NA
HCV	25 (9.4%)	34 (7.5%)	5 (3.6%)	NA
MASLD	142 (53.2%)	242 (53.2%)	113 (81.3%)	NA
ALD	51 (19.1%)	76 (16.7%)	1 (0.7%)	NA
Others	39 (14.6%)	96 (21.1%)	3 (2.2%)	NA
BCLC-Stage*				
0-A	127 (52.3%)	NA	NA	NA
B	56 (23.0%)	NA	NA	NA
C-D	59 (24.3%)	NA	NA	NA
Unknown	1 (0.4%)	NA	NA	NA
PNPLA3 rs738409				
CC	29 (10.9%)	60 (13.2%)	38 (27.3%)	37 (33.3%)
CG	92 (34.5%)	187 (41.1%)	61 (43.9%)	52 (46.8%)
GG	146 (54.7%)	208 (45.7%)	40 (28.8%)	22 (19.8%)
MBOAT7 rs641738				
CC	93 (34.8%)	159 (34.9%)	55 (39.6%)	44 (39.6%)
CT	124 (46.4%)	219 (48.1%)	72 (51.8%)	54 (48.6%)

TT	50 (18.7%)	77 (16.9%)	12 (8.6%)	13 (11.7%)
HSD17B13 rs72613567				
TT	234 (87.6%)	356 (78.2%)	107 (77.0%)	79 (71.2%)
TAT	32 (12.0%)	91 (20.0%)	26 (18.7%)	27 (24.3%)
TATA	1 (0.4%)	8 (1.8%)	6 (4.3%)	5 (4.5%)
TM6SF2 rs58542926				
CC	236 (88.4%)	405 (89.0%)	123 (88.5%)	99 (89.2%)
CT	31 (11.6%)	47 (10.3%)	16 (11.5%)	12 (10.8%)
TT	0	3 (0.7%)	0	0

* Only cirrhotic HCC patients were assessed using the BCLC-stage. Abbreviations: ALD, alcoholic liver disease; HBV, hepatitis B virus; HCV, hepatitis C virus; HCC, hepatocellular carcinoma; MASLD, metabolic dysfunction-associated steatotic liver disease; NCLD, non-cirrhotic liver disease; PNPLA3, Patatin-like phospholipase domain-containing protein 3; MBOAT7, Membrane-bound O-acyltransferase domain-containing protein 7; HSD17B13, Hydroxysteroid 17-beta dehydrogenase 13; TM6SF2, Transmembrane 6 superfamily member 2. BCLC, Barcelona Clinic Liver Cancer Staging System.

Table S2. Power analysis for individual SNPs and GRS in MASLD-related liver disease

Comparison	Cirrhotic HCC vs. Cirrhosis	VAF	Minimal detectable OR (80% power)	α
PNPLA3	142 vs. 242	0.71	OR of ≥2.07 (or ≤0.54 for protective effects)	0.05
MBOAT7	142 vs. 242	0.42	OR of ≥1.81 (or ≤0.53 for protective effects)	0.05
TM6SF2	142 vs. 242	0.05	OR of ≥2.92 (or ≤0.03 for protective effects)	0.05
HSD17B13	142 vs. 242	0.10	OR of ≥2.31 (or ≤0.24 for protective effects)	0.05
GRS 6-8 vs. 0-2	28 vs. 40	0.55	OR of ≥5.09 (or ≤0.23 for protective effects)	0.05
	Cirrhotic HCC vs. NCLD			
PNPLA3	142 vs. 113	0.49	OR of ≥2.05 (or ≤0.49 for protective effects)	0.05
MBOAT7	142 vs. 113	0.35	OR of ≥2.05 (or ≤0.45 for protective effects)	0.05
TM6SF2	142 vs. 113	0.05	OR of ≥3.57	0.05
HSD17B13	142 vs. 113	0.16	OR of ≥2.35 (or ≤0.29 for protective effects)	0.05
GRS 4 vs. 0-2	51 vs. 65	0.65	OR of ≥3.70 (or ≤0.35 for protective effects)	0.05
GRS 5 vs. 0-2	49 vs. 39	0.41	OR of ≥3.42 (or ≤0.25 for protective effects)	0.05
GRS 6-8 vs. 0-2	28 vs. 25	0.08	OR of ≥7.82	0.05
	Cirrhosis vs. NCLD			
PNPLA3	242 vs. 113	0.49	OR of ≥1.91 (or ≤0.52 for protective effects)	0.05
MBOAT7	242 vs. 113	0.35	OR of ≥1.92 (or ≤0.49 for protective effects)	0.05
TM6SF2	242 vs. 113	0.05	OR of ≥3.29 (or ≤0.06 for protective effects)	0.05
HSD17B13	242 vs. 113	0.16	OR of ≥2.20 (or ≤0.35 for protective effects)	0.05
GRS 4 vs. 0-2	76 vs. 65	0.65	OR of ≥3.10 (or ≤0.38 for protective effects)	0.05
GRS 5 vs. 0-2	81 vs. 39	0.41	OR of ≥3.02 (or ≤0.29 for protective effects)	0.05
GRS 6-8 vs. 0-2	40 vs. 25	0.08	OR of ≥7.03	0.05

Abbreviations: HCC, hepatocellular carcinoma; NCLD, non-cirrhotic liver disease; PNPLA3, Patatin-like phospholipase domain-containing protein 3; MBOAT7, Membrane-bound O-acyltransferase domain-containing protein 7; HSD17B13, Hydroxysteroid 17-beta dehydrogenase 13; TM6SF2, Transmembrane 6 superfamily member 2; OR, odds ratio; VAF, variant allele frequency; GRS, genetic risk score.

Table S3. The impact of the 4 individual SNPs in MASLD-related liver disease

	Cirrhotic HCC vs. cirrhosis		Cirrhotic HCC vs. NCLD		Cirrhosis vs. NCLD	
SNPs	OR 95% CI (Adjusted)	P	OR 95% CI (Adjusted)	P	OR 95% CI (Adjusted)	P
PNPLA3						
CC	Reference	NA	Reference	NA	Reference	NA
CG	1.334 (0.502-3.545)	0.563	3.550 (1.112-11.33)	0.032	3.043 (1.469-6.303)	0.003
GG	2.574 (0.986-6.722)	0.053	13.12 (4.076-42.24)	<0.001	6.401 (3.031-13.52)	<0.001
MBOAT7						
CC	Reference	NA	Reference	NA	Reference	NA
CT	0.653 (0.388-1.100)	0.109	0.579 (0.274-1.221)	0.151	1.065 (0.626-1.812)	0.815
TT	1.050 (0.551-2.000)	0.883	2.424 (0.790-7.435)	0.122	2.211 (0.884-5.532)	0.090
HSD17B13						
TT	Reference	NA	Reference	NA	Reference	NA
TAT	0.636 (0.323-1.253)	0.191	0.443 (0.169-1.163)	0.098	0.664 (0.359-1.228)	0.191
TATA	0.289 (0.022-3.848)	0.347	0.027 (0.001-0.625)	0.024	0.119 (0.023-0.605)	0.010
TM6SF2						
CC	Reference	NA	Reference	NA	Reference	NA
CT	1.172 (0.544-2.527)	0.685	0.746 (0.268-2.071)	0.573	0.702 (0.316-1.559)	0.385
TT	NA	NA	NA	NA	NA	NA

The odds ratio (OR) was adjusted for age, gender, BMI, diabetes and ethnicity. Abbreviations: MASLD, metabolic dysfunction-associated steatotic liver disease; NCLD, non-cirrhotic liver disease; PNPLA3, Patatin-like phospholipase domain-containing protein 3; MBOAT7, Membrane-bound O-acyltransferase domain-containing protein 7; HSD17B13, Hydroxysteroid 17-beta dehydrogenase 13; TM6SF2, Transmembrane 6 superfamily member 2; OR, odds ratio; CI, confidence interval; NA, not available.

Table S4. The impact of the 4 SNPs on the development of HCC compared to cirrhosis in Viral and ALD

	HBV+HCV		ALD	
SNPs	OR 95% CI (Adjusted)	P	OR 95% CI (Adjusted)	P
PNPLA3				
CC	Reference	NA	Reference	NA
CG	0.914 (0.238-3.515)	0.895	0.612 (0.107-3.497)	0.581
GG	0.934 (0.421-2.071)	0.866	0.276 (0.042-1.804)	0.179
MBOAT7				
CC	Reference	NA	Reference	NA
CT	2.099 (0.655-6.730)	0.212	1.722 (0.700-4.235)	0.237
TT	0.996 (0.492-2.016)	0.991	1.841 (0.492-6.887)	0.365
HSD17B13				
TT	Reference	NA	Reference	NA
TAT	0.619 (0.163-2.349)	0.481	0.136 (0.034-0.536)	0.004
TATA	NA	NA	NA	NA
TM6SF2				
CC	Reference	NA	Reference	NA
CT	0.650 (0.160-2.631)	0.546	2.532 (0.706-9.079)	0.154
TT	NA	NA	NA	NA

The odds ratio (OR) was adjusted for age, gender, BMI, and ethnicity. Abbreviations: HCC, hepatocellular carcinoma; PNPLA3, Patatin-like phospholipase domain-containing protein 3; MBOAT7, Membrane-bound O-acyltransferase domain-containing protein 7; HSD17B13, Hydroxysteroid 17-beta dehydrogenase 13; TM6SF2, Transmembrane 6 superfamily member 2; OR, odds ratio; CI, confidence interval; ALD, alcoholic liver disease; HBV, hepatitis B virus; HCV, hepatitis C virus; NA, not available.

Table S5. The impact of the 4 SNPs on the development of cirrhosis compared to NCLD in Viral

SNPs	OR 95% CI (Adjusted)	P
PNPLA3		
CC	Reference	NA
CG	1.152 (0.144-9.226)	0.894
GG	0.743 (0.223-2.478)	0.629
MBOAT7		
CC	Reference	NA
CT	0.727 (0.149-3.544)	0.693
TT	2.671 (0.766-9.312)	0.123
HSD17B13		
TT	Reference	NA
TAT	0.571 (0.058-5.605)	0.631
TATA	NA	NA
TM6SF2		
CC	Reference	NA
CT	1.650 (0.225-12.08)	0.622
TT	NA	NA

The odds ratio (OR) was adjusted for age, gender, BMI, and ethnicity. Abbreviations: Viral, HBV+HCV; HBV, hepatitis B virus; HCV, hepatitis C virus; NCLD, non-cirrhotic liver disease; PNPLA3, Patatin-like phospholipase domain-containing protein 3; MBOAT7, Membrane-bound O-acyltransferase domain-containing protein 7; HSD17B13, Hydroxysteroid 17-beta dehydrogenase 13; TM6SF2, Transmembrane 6 superfamily member 2; OR, odds ratio; CI, confidence interval; NA, not available.

Table S6. Comparison of the performance of GRS and *PNPLA3* in MASLD-related liver disease

Cirrhotic HCC vs. cirrhosis	*PNPLA3*	GRS	P
AUC	0.587 (0.528-0.646)	0.564 (0.503-0.624)	0.371
Cirrhotic HCC vs. NCLD			
AUC	0.739 (0.676-0.802)	0.729 (0.667-0.791)	0.721
Cirrhosis vs. NCLD			
AUC	0.661 (0.600-0.722)	0.671 (0.612-0.729)	0.698

Abbreviations: HCC, hepatocellular carcinoma; NCLD, non-cirrhotic liver disease; PNPLA3, Patatin-like phospholipase domain-containing protein 3; GRS, genetic risk score; AUC, area under the curve.

Table S7. Quality control in chemokine measurements

	LL	Kit control			Healthy control			HCV control			
Markers	pg/mL	Mean	SD	CV	Mean	SD	CV	Mean	SD	CV	Excluded
IL-10	2.14	243.42	14.36	0.06	6.41	7.40	**1.15**	10.62	7.47	**0.70**	**Yes**
CCL19	64.68	1260.62	47.73	0.04	162.71	127.95	**0.79**	272.15	79.18	0.29	**Yes**
CCL7	101.18	1277.89	139.46	0.11	680.26	452.85	**0.67**	849.06	420.77	**0.50**	**Yes**
IL-6	2.99	888.15	142.71	0.16	12.56	7.90	**0.63**	42.37	30.72	**0.73**	**Yes**
CXCL5	548.23	9945.45	647.44	0.07	1116.58	655.98	**0.59**	2047.98	716.24	**0.35**	**Yes**
CCL20	2.58	61.31	4.94	0.08	3.61	1.78	**0.49**	3.61	1.78	**0.49**	**Yes**
GM-CSF	2.60	1980.06	53.83	0.03	36.76	15.48	**0.42**	45.70	17.31	**0.38**	**Yes**
CCL26	12.87	302.65	10.24	0.03	127.06	47.57	**0.37**	166.59	53.02	**0.32**	**Yes**
IL-4	13.33	213.72	12.08	0.06	109.67	34.66	**0.32**	147.27	36.96	0.25	**Yes**
CXCL13	4.32	37.51	4.16	0.11	22.21	6.83	**0.31**	21.65	5.01	0.23	**Yes**
IL-16	13.02	7738.90	259.80	0.03	185.13	55.66	**0.30**	226.87	66.13	0.29	**Yes**
CCL17	9.49	21.28	20.42	**0.96**	159.35	17.65	0.11	255.95	18.62	0.07	**Yes**
CCL21	242.03	1821.61	531.36	0.29	33069.91	1098.28	0.03	40912.82	24358.54	**0.60**	**Yes**
IL-1b	0.48	206.33	9.60	0.05	6.22	1.76	0.28	7.08	1.52	0.21	No
CCL1	20.70	191.86	13.07	0.07	131.65	34.59	0.26	158.12	34.05	0.22	No
CXCL9	8.38	98.77	4.27	0.04	42.17	10.60	0.25	77.38	18.03	0.23	No
CXCL12	25.17	1955.16	168.90	0.09	1045.00	240.36	0.23	1103.27	259.22	0.23	No
CCL25	50.32	3603.70	312.34	0.09	1065.22	241.62	0.23	1346.68	262.25	0.19	No
MIF	159.76	10005.15	230.49	0.02	10840.08	2218.65	0.20	14817.51	2754.80	0.19	No
TNFα	1.10	347.23	68.25	0.20	11.28	2.26	0.20	16.61	3.02	0.18	No
CCL23	4.85	604.69	32.13	0.05	215.83	43.10	0.20	283.69	46.48	0.16	No
CXCL6	11.53	184.53	7.96	0.04	72.58	13.17	0.18	87.01	20.97	0.24	No
CCL13	3.60	59.62	7.49	0.13	52.02	9.30	0.18	68.72	9.80	0.14	No
CXCL1	81.77	2956.37	229.88	0.08	655.89	112.40	0.17	770.74	159.96	0.21	No
CXCL10	22.90	199.71	17.43	0.09	132.16	21.97	0.17	336.23	32.37	0.10	No
CCL22	3.88	517.74	96.51	0.19	508.83	81.96	0.16	930.67	56.41	0.06	No
CXCL16	5.34	292.68	18.22	0.06	269.33	40.19	0.15	333.68	24.57	0.07	No
CCL2	0.47	26.46	1.26	0.05	59.25	8.83	0.15	82.78	12.79	0.15	No
CCL15	4.18	2166.14	126.13	0.06	3198.55	439.00	0.14	4727.34	395.57	0.08	No
IFNγ	0.54	78.40	4.53	0.06	5.45	0.65	0.12	8.23	0.85	0.10	No
CXCL11	0.21	14.82	0.23	0.02	12.14	1.44	0.12	26.82	2.06	0.08	No
CCL8	0.79	36.03	4.62	0.13	64.86	7.62	0.12	86.03	10.61	0.12	No
CCL27	10.25	277.75	29.38	0.11	573.43	66.40	0.12	1083.38	77.33	0.07	No
CXCL8	1.02	130.98	6.61	0.05	13.68	1.57	0.11	28.07	1.01	0.04	No
CCL3	0.59	365.30	25.79	0.07	11.77	1.33	0.11	14.92	1.41	0.09	No
CCL24	2.74	98.71	9.80	0.10	230.11	24.68	0.11	382.93	25.93	0.07	No
CCL11	5.53	238.83	30.01	0.13	69.90	6.54	0.09	92.49	6.31	0.07	No
CXCL2	21.45	311.51	27.39	0.09	325.94	23.40	0.07	435.24	19.75	0.05	No
CX3CL1	21.93	352.78	15.75	0.04	21.93	0.00	0.00	21.93	0.00	0.00	No

IL-2	5.52	1586.33	76.91	0.05	5.52	0.00	0.00	5.96	0.76	0.13	No
MMP2	1103.20	NA	NA	NA	13965.79	1252.05	0.09	14517.33	2589.41	0.18	No
MMP3	1158.49	NA	NA	NA	5637.94	760.34	0.13	8427.49	2315.51	0.27	No
Pentraxin-3	47.93	NA	NA	NA	8288.89	125.51	0.02	8786.67	564.51	0.06	No

Abbreviations: LL, lower limit detection; SD, standard deviation; CV, coefficient of variation.

Table S8. Cytokine levels in MASLD-related liver disease with GRS 0-4 and GRS 5-8

	Cirrhotic HCC				Cirrhosis			
Cytokine	GRS 0-4	GRS 5-8	P	FDR	GRS 0-4	GRS 5-8	P	FDR
IL-1b	4.425	3.84	0.108	0.233	4.36	4.91	0.165	0.544
CCL1	100.5	92.11	0.060	0.168	96.79	124.7	**0.034**	0.364
CXCL9	82.93	63.11	**0.016**	0.084	73.52	80.83	0.688	0.935
CXCL12	1164	1136	0.779	0.808	1191	1287	0.296	0.544
CCL25	977.8	833.2	0.102	0.233	961	1266	0.078	0.544
MIF	13366	11480	0.254	0.374	13320	16155	0.224	0.544
TNFα	13.89	11.41	**0.023**	0.092	13.13	14.34	0.125	0.544
CCL23	296.7	283.5	0.625	0.729	237.5	220.1	0.671	0.935
CXCL6	92.82	82.03	0.533	0.678	82.03	116.1	0.110	0.544
CCL13	33.99	25.38	**0.042**	0.147	39.85	39.22	0.826	0.964
CXCL1	451.8	373.6	0.050	0.156	536.4	495.2	0.874	0.973
CXCL10	248.3	168.2	**0.016**	0.084	170.8	211.7	0.189	0.544
CCL22	361.9	321.6	0.724	0.780	414.8	447.2	0.296	0.544
CXCL16	342.7	300.6	**0.018**	0.084	341.8	363.5	0.426	0.702
CCL2	72.5	61.6	**0.018**	0.084	69.49	73.44	0.780	0.957
CCL15	4065	3520	0.203	0.339	4058	3769	0.701	0.935
IFNγ	4.17	2.33	**0.0008**	**0.011**	3.97	3.885	0.938	0.973
CXCL11	9.23	6.48	0.087	0.221	9.1	9.22	0.223	0.544
CCL8	60.33	43.04	**0.0007**	**0.011**	64.86	51.23	0.472	0.734
CCL27	999.7	906.6	0.206	0.339	1066	1140	0.246	0.544
CXCL8	30.2	33.09	0.592	0.721	23.28	30.34	**0.039**	0.364
CCL3	9.8	9.15	0.197	0.339	8.56	8.925	0.987	0.987
CCL24	203	226.5	0.319	0.425	233.9	234.5	0.917	0.973
CCL11	74.63	71.53	0.273	0.382	85.31	85.78	0.311	0.544
CXCL2	146.5	126.3	0.172	0.339	151.6	163.5	0.786	0.957
MMP2	40807	45334	0.245	0.374	34145	51751	**0.0006**	**0.017**
MMP3	11316	10877	0.695	0.778	11122	12643	0.285	0.544
Pentraxin-3	28592	29020	0.846	0.846	19234	23828	0.143	0.544

IL2 and CX3CL1 were excluded because most samples reached the lower limit of detection. Abbreviations: HCC, hepatocellular carcinoma; MASLD, metabolic dysfunction-associated steatotic liver disease; FDR, false discovery rate.
