# Supplementary material for: Genetic risk score correlates with immune profile and risk of HCC and cirrhosis development in Hispanics with MASLD
Source: JHEP Rep. 2025 Jul 2;7(10):101508. doi: 10.1016/j.jhepr.2025.101508 (PMC12455133; doi:10.1016/j.jhepr.2025.101508)
Supplement: Multimedia component 2 [file mmc2.docx]

**JHEP Reports**

**CTAT methods**

Tables for a “Complete, Transparent, Accurate and Timely account” (CTAT) are now mandatory for all revised submissions. The aim is to enhance the reproducibility of methods.

- Only include the parts relevant to your study
- Refer to the CTAT in the main text as ‘Supplementary CTAT Table’
- Do not add subheadings
- Add as many rows as needed to include all information
- Only include one item per row

**If the CTAT form is not relevant to your study, please outline the reasons why:**

|  |
| --- |

- 1. **Antibodies**

| **Name** | **Citation** | **Supplier** | **Cat no.** | **Clone no.** |
| --- | --- | --- | --- | --- |
|  |  |  |  |  |

- 1. **Cell lines**

| **Name** | **Citation** | **Supplier** | **Cat no.** | **Passage no.** | **Authentication test method** |
| --- | --- | --- | --- | --- | --- |
|  |  |  |  |  |  |

- 1. **Organisms**

| **Name** | **Citation** | **Supplier** | **Strain** | **Sex** | **Age** | **Overall n number** |
| --- | --- | --- | --- | --- | --- | --- |
|  |  |  |  |  |  |  |

- 1. **Sequence based reagents**

| **Name** | **Sequence** | **Supplier** |
| --- | --- | --- |
| PNPLA3 rs738409 | Assay ID C______7241_10 | ThermoFisher |
| MBOAT7 rs641738 | Assay ID C___8716820_10 | ThermoFisher |
| HSD17B13 rs72613567 | Assay ID AN7D39Z | ThermoFisher |
| TM6SF2 rs58542926 | Assay ID C__89463510_10 | ThermoFisher |

- 1. **Biological samples**

| **Description** | **Source** | **Identifier** |
| --- | --- | --- |
| Serum samples | Patients with liver disease | ESCALON consortium |

- 1. **Deposited data**

| **Name of repository** | **Identifier** | **Link** |
| --- | --- | --- |
|  |  |  |

- 1. **Software**

| **Software name** | **Manufacturer** | **Version** |
| --- | --- | --- |
| Rstudio | Posit, PBC | 4.1.1 |
| SPSS | IBM | 28.0.1.0 |
| StepOne | Applied Biosystems | 2.3 |
| GraphPad Prism | GraphPad Software, LLC | 8.0.2 |

- 1. **Other (*e.g*. drugs, proteins, vectors etc.)**

| Bio-Plex Human Cytokine 40-Plex panel | Bio-Rad |  |
| --- | --- | --- |
| Single-Plex kits for Pentraxin-3, MMP-2, and MMP-3 | Bio-Rad |  |
| Genotyping master mix | Applied Biosystems |  |

- 1. **Please provide the details of the corresponding methods author for the manuscript:**

| José D Debes, j.debes@erasmusmc.nl, debes003@umn.edu |
| --- |

**2.0 Please confirm for randomised controlled trials all versions of the clinical protocol are included in the submission. These will be published online as supplementary information.**

|  |
| --- |
